# *Galleria mellonella* Invertebrate Model Mirrors the Pathogenic Potential of *Mycoplasma alligatoris* within the Natural Host

**DOI:** 10.1155/2024/3009838

**Published:** 2024-03-22

**Authors:** Alexandra M. Burne, Lauren J. Richey, Trenton R. Schoeb, Mary B. Brown

**Affiliations:** ^1^Department of Infectious Disease and Immunology, College of Veterinary Medicine, University of Florida, Gainesville, FL 32608, USA; ^2^Comparative Pathology Services, Tufts University, Boston, MA 02155, USA; ^3^Program in Immunology, Heersink School of Medicine, University of Alabama Birmingham, Birmingham, AL 35294, UK

## Abstract

Most mycoplasmal infections result in chronic, clinically silent disease. In direct contrast, *Mycoplasma alligatoris* elicits a fulminant, multisystem disease in the natural host, *Alligator mississippiensis* (American alligator). The goals of the study were to better understand the disease in the natural host and to determine if the invertebrate model *G. mellonella* could serve as a surrogate alternate host. The survival of alligators infected intratracheally was dose dependent (*p*=0.0003), ranging from no mortality (10^2^ CFU) to 100% mortality (10^8^ CFU), with 60% mortality at the 10^4^ and 10^5^ CFU infectious dose. Microbial load in blood, joints, and brain was dose dependent, regardless of whether alligators were infected intratracheally or intravenously (*p*  < 0.002). Weight loss was similarly impacted (*p*  < 0.001). Experimental infection of the invertebrate *Galleria mellonella* mirrored the result in the natural host. In a dose response infection study, both larval survival curves and successful pupation curves were significantly different (*p* ≤ 0.0001) and dose dependent. Infected insects did not emerge as moths (*p*  < 0.0001). Here, we describe the first study investigating *G. mellonella* as a surrogate model to assess the pathogenic potential of *M. alligatoris*. *G. mellonella* survival was dose dependent and impacted life stage outcome.

## 1. Introduction

Mycoplasmas are important pathogens of human and veterinary medicine, causing both acute and chronic infections [1–10]. Mycoplasmas have minimal genomes, ranging from half a million to two million base pairs, making them the smallest free-living organisms capable of self-replication outside of their host [11–14]. Because of genome reduction, mycoplasmas have lost their cell wall as well as many biosynthetic capabilities, resulting in reliance upon the host to provide additional nutrients required for growth [12, 14–16]. Aside from hemoplasmas, which specifically target and infect erythrocytes [17–20], the primary site of colonization for mycoplasmas is mucosal surfaces [10, 21]. Infection with most *Mycoplasma* spp. is characterized by low mortality but high morbidity, most commonly presenting as a chronic, and often clinically silent, disease [2, 4–7, 9, 22–26]. *Mycoplasma alligatoris* is one the few species of *Mycoplasma* that elicit fulminant disease in their host [27–29]. *M. alligatoris* was first identified in captive American alligators in a Florida zoological park in 1995. Within a 10-day period of the outbreak, nine of 74 captive bull alligators (300–350 kg, >30 years old) died acutely because of the infection. Over a 6 months period, 51 other animals died or were euthanized, for an 81% mortality rate. Clinical signs included anorexia, lethargy, muscle weakness, paraparesis, bilateral white ocular discharge, and various degrees of periocular, facial, cervical, and limb edema. The major necropsy findings included pericarditis, myocarditis, polyarthritis, and pneumonia. In the U.S., alligator farming is a $77 million industry and represents >54% of miscellaneous aquaculture sales [30, 31]. Therefore, this pathogen has the potential to impact not only wild populations but also farmed alligators.

Current methods to determine virulence potential and host-pathogen interactions of mycoplasmas have relied on (i) *in vivo* experimental infections of the natural or alternative hosts [32–35] or on (ii) *in vitro* infections [21, 36–40]. Infections of the natural host, especially for reptiles, can be challenging but have been successfully used to confirm that both *M. alligatoris* [27, 41] and *M. agassizii* [42, 43] are etiologic agents of disease in alligators and tortoises, respectively. However, high throughput *in vivo* screening of clinical isolates or mutants to determine differences in pathogenicity is neither fiscally nor ethically manageable in reptiles. *In vitro* assays have focused on the use of cell lines to screen a large number of *Mycoplasma* spp. for pathogenic potential and to understand cellular and molecular interactions [10, 21, 36, 37, 39, 40, 44]. However, cell lines lack a functioning immune system which is a key component of mycoplasmal disease [1, 2, 45–48]. All of these methods have major limitations when considering *Mycoplasma* spp. from reptilian hosts as most have a strict temperature growth restriction and cannot be cultivated above 30°C. As most commercially available cell lines are of mammalian origin and propagated at 37°C, they are not applicable for studying mycoplasmas isolated from poikilotherms. The commercial availability of reptiles for *in vivo* infection studies is virtually nonexistent, and many reptilian species are threatened or endangered [49]. For larger reptiles such as the Crocodilians, housing, feeding, and handling can be challenging for most Animal Care Services.

*Mycoplasma* spp. are increasingly detected from a range of wildlife hosts [50, 51]. Sampling of wild caprine, avian, rodent, and marine mammal hosts has identified the emergence of novel *Mycoplasma* species as well as known pathogens of domestic animals [23, 52–61]. Although many of the new species have been cultured and therefore theoretically could be amenable to infection studies, the subgroup of nonculturable hemotropic mycoplasmas [18, 19, 50] poses a unique challenge for pathogenicity studies. The emergence of *Mycoplasma ovipneumoniae* in big horn sheep and *Mycoplasma gallisepticum* in wild house finch populations provide examples of overcoming some of the challenges of studying mycoplasmal disease in wildlife species by use of alternative hosts [62–64]. However, even when clinical isolates have been obtained, there are still difficulties in determining association the of disease etiology, pathogenicity, and virulence potential in wild hosts that are ecologically at risk and/or understudied (e.g., marine mammals). Therefore, there is a critical need for an alternative model that has a functioning immune system, can be cultivated between 25 and 37°C, and can be used for screening of *Mycoplasma* species, clinical isolates, and mutants.

Invertebrate models using *Caenorhabditis elegans*, *Drosophila melanogaster*, and *Galleria mellonella* to determine pathogenic potential and specific virulence factors of both bacteria and fungi are an increasingly recognized alternative to vertebrate testing [65–70]. All of these invertebrate models share common innate immune system features with vertebrates, including toll-like receptors, microbial killing pathways, C-lectins, and apoptotic pathways [67, 71–75]. Additionally, invertebrate models are highly economical, commercially available, and allow for a robust sample size, allowing researchers to conduct high throughput screening of mutant libraries, strains, and clinical isolates [71, 73, 76–82]. In a recent review [83], 72% of the bacterial species studied were Gram-negatives, including species in the genera *Pseudomonas*, *Acinetobacter*, *Klebsiella*, *Escherichia*, *Burkholderia*, and *Campylobacter;* Gram-positive bacteria comprised 26% of the studied genera, including *Staphylococcus*, *Enterococcus*, *Bacillus*, *Listeria*, and *Streptococcus*. Intracellular bacteria including the genera *Coxiella* and *Francisella* also have tested in *G. mellonella* [71]. Finally, the *G. mellonella* has been applied to a number of fungal pathogens such as *Candida*, *Aspergillus*, and *Cryptococcus* [84].

Importantly, unlike *C. elegans* and *D. melanogaster*, *G. mellonella* has a wide temperature range and can be propagated between 15 and 37°C, making them a suitable alternative model for temperature-restricted bacteria and fungi [73, 85, 86]. Therefore, *G. mellonella* was selected to develop and confirm the applicability of invertebrates as a model system for *M. alligatoris*. Here we report the characterization of the *G. mellonella* infection model for *M. alligatoris* and comparison of the invertebrate model system with experimental dose response infections in the natural host, *Alligator mississippiensis*.

## 2. Materials and Methods

### 2.1. Source, Growth, and Infectious Dose Confirmation of *M. alligatoris*

*M. alligatoris* ATCC 700619 strain A21JP2 was used for all studies. This is the type strain and was isolated from the joint of one of a group of captive alligators affected by a fatal epizootic disease in St. Johns County, Florida, in 1995 and 1996 [29, 87]. SP4 broth and agar [88] were used for cultivation. Because of temperature restrictions of *M. alligatoris*, all cultures were incubated at 30°C. The same frozen stock culture was used for all experimental infections of alligators. For infection of *G. mellonella*, *M. alligatoris* was grown in SP4 broth and the inoculums were standardized to low (10^2^), medium (10^4^), and high (10^6^) prior to use for infection. The same frozen stock culture was used for both survival and bacterial burden inoculations. For stock cultures, both the color change units (CCU) and colony forming units (CFU) were confirmed by dilution in SP4 broth and plating on SP4 agar prior to freezing. At the time of infection, the frozen stock was thawed, and the infectious dose used was confirmed by dilution in SP4 broth and plating on SP4 agar. Broth and agar dilutions were performed in duplicate and incubated at 30°C for 4 days.

### 2.2. Animal Care and Housing (Figure 1)

**Figure 1:**
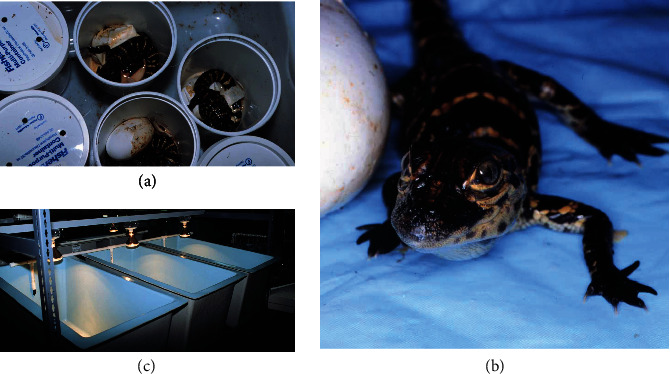
Husbandry of alligators. (a) Alligator eggs were incubated at 33°C. (b) Hatchlings were maintained in large plastic containers (c) tilted at an angle to provide approximately 7 cm water at the lower end and a dry area for basking at the higher end. Heat lamps provided 12-hr on/off cycles of light.

Animals were hatched (Figures 1(a) and 1(b) and maintained at the Florida Caribbean Science Center, United States Geological Survey, Biological Resources Division, Gainesville, FL, until used in the experiments described below. For infection studies, hatchlings were transferred to the infectious disease unit of the University of Florida Animal Resources and were acclimated for 1 week before inoculation. Hatchlings were identified with metal tags in the rear footwebs. Animals were maintained (Figures 1(b) and 1(c)) in large plastic containers (113–189 L) that were tilted at an angle to provide approximately 7 cm of water at the lower end and a dry area for basking at the higher end. Heat lamps provided 12-hr on/off cycles of light. Ambient air temperature ranged from 21 to 23°C. Animals were fed a commercial pelleted alligator diet (Burris Mill and Feed, Inc., Franklinton, LA) three times weekly. Animals were euthanized with an intracardiac or an intravenous (IV) injection in the occipital sinus of Beuthanasia®-D (Schering–Plough Animal Health Corporation, Kenilworth, NJ).

### 2.3. Intratracheal (IT) Dose Response Study

Alligator eggs were purchased from a reference site (Agrifos, Tampa, Florida) and were artificially incubated at approximately 33°C. After hatching, 12-week-old animals (*n* = 63 total infected) were inoculated via IT administration of 10^1^, 10^2^, 10^3^, 10^4^, 10^5^, 10^6^, or 10^7^ CFU of late log phase *M. alligatoris* in 20 *μ*L SP4 broth (*n* = 9 per infection dose). Controls (*n* = 7) received sterile SP4 broth. Animals in each group were necropsied at 2 (*n* = 4, each infectious dose; *n* = 3, controls) or 4 (*n* = 5, each infectious dose; *n* = 4, controls) weeks postinfection (PI). Two animals (1 in 10^2^ and 1 in 10^8^) were euthanized prior to the 2-week necropsy. Blood, lung, and one-half of the brain were collected for quantitative culture. Samples of blood, brain, lung, tracheal fluid, and joint fluid were diluted and plated in modified SP4 and were incubated at 30°C.

### 2.4. Intravenous (IV) Dose Response Studies

An IV dose response experiment, similar to the IT infection study, was performed using 22-week-old animals (*n* = 21, total infected; *n* = 5, control) from a reference lake (Rockefeller National Wildlife Refuge, Grand Chenier, LA). *M. alligatoris* was administered intravenously via injection of 100 *μ*L into the occipital sinus at doses of 10^2^, 10^4^, 10^6^, or 10^8^ CFU (*n* = 5, for each dose group except 10^8^ CFU, which had *n* = 6). Controls (*n* = 5) received sterile SP4 broth. The inoculation doses were confirmed by dilution and plating. Animals were euthanized if they showed signs of severe illness. Criteria for euthanasia included severe lameness of multiple limbs, greatly reduced responses to external stimuli, or a weight loss greater than 20% of the original body weight. All animals were necropsied on either (i) the day of spontaneous death, (ii) the day the criteria were met for euthanasia, or (iii) at the end of the study, 28 days postinoculation. At necropsy, blood, brain, and swabs of stifle and elbow joints were serially diluted in SP4 broth and incubated at 30°C for 4 days to detect CCU.

### 2.5. Intravenous (IV) Infection of Alligators (*n* = 18) with a Single Dose (10^6^ CFU) of *M. alligatoris*

In the preliminary IT and IV dose response studies, an infectious dose of 10^6^ CFU of *M. alligatoris* resulted in consistent clinical disease and lesions without significant mortality. Regardless of the route of infection, consistent colonization of the blood and joints was observed. In animals infected IT, *M. alligatoris* was recovered from blood but not trachea. Therefore, the IV route was chosen for the more intensive single dose study. Daily assessments of appetite and general body condition were made. Limb swelling and lameness were visually assessed daily both visually and by caliper measurement. Body weights were obtained weekly from all animals on days (*D*) 0, 7, 14, 21, and 28 PI. Because there was some evidence of recovery from both weight loss and lameness in the preliminary IT and IV infection studies, we did not include weight loss as a criterion for euthanasia. However, any animal that became ataxic, unresponsive to external stimuli, or that developed a head tilt was euthanized before D28 PI. Controls (*n* = 5) received sterile SP4 broth.

### 2.6. Histopathology

Because the main purpose of the initial IT and IV dose experiments was to determine the optimal dose and route for a more intensive infection study, a less intensive necropsy was performed on these animals. A subset of lung samples (*n* = 4) from each infection dose as well as brain, elbow, and knee joints of all animals were fixed in alcoholic formalin, trimmed, routinely processed, embedded in paraffin, and sectioned at 4 *μ*M.

For the 28 days infection study of alligators receiving 10^6^ CFU via the IV route, full necropsies were performed. Samples of brain, thoracic and pelvic limbs, thymus, spleen, and any tissues with gross lesions were fixed in 10% neutral buffered formalin. After fixation for at least 24 hr, each foot was removed from the limb just above the carpus or tarsus and was processed to include the carpal/tarsal, metacarpalphalangeal, and interphalangeal joints. The joints were kept intact and were not opened for culture to preserve the integrity of the joint. Tissues were embedded in paraffin, sectioned at 5 *µ*M, and stained with hematoxylin and eosin.

The slides were coded so that the pathologist was blinded to the treatment group and animal identity. The scoring criteria are provided in Table 1. Brain lesions were assessed based on severity of encephalitis and meningitis. Joint lesions in the tarsi and carpi were scored based on cartilage erosion, subchondral bone lesions, joint space exudate, and synovitis. An index score was then calculated by dividing the total lesion score by the total possible points. For example, an animal that had severe meningitis and mild encephalitis would have a brain index of 4/6 or 0.67. Both a brain lesion severity index and an index for the combined scores of all four tarsal and carpal joints were determined. Animals that died before the end of the study at 28 days were excluded from histologic comparisons so that lesion severity would be judged based on the same chronicity of infection.

### 2.7. *G. mellonella* Infection

**Figure 2:**
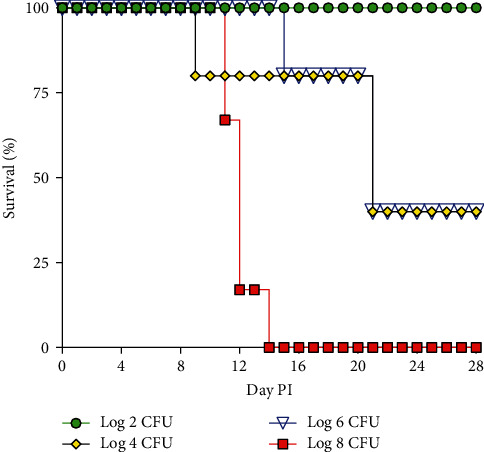
Survival curve for 22-week-old alligators inoculated intravenously with *M. alligatoris*. For infectious dose 10^2^, 10^4^, and 10^6^ groups, *n* = 5; for 10^8^ group, *n* = 6. No mortality was observed in controls (*n* = 5), data are not shown. Survival curves were analyzed by Kaplan–Meier and log-rank Mantel–Cox test for significance. Survival curves were significantly different, *p*=0.0003.

**Figure 3:**
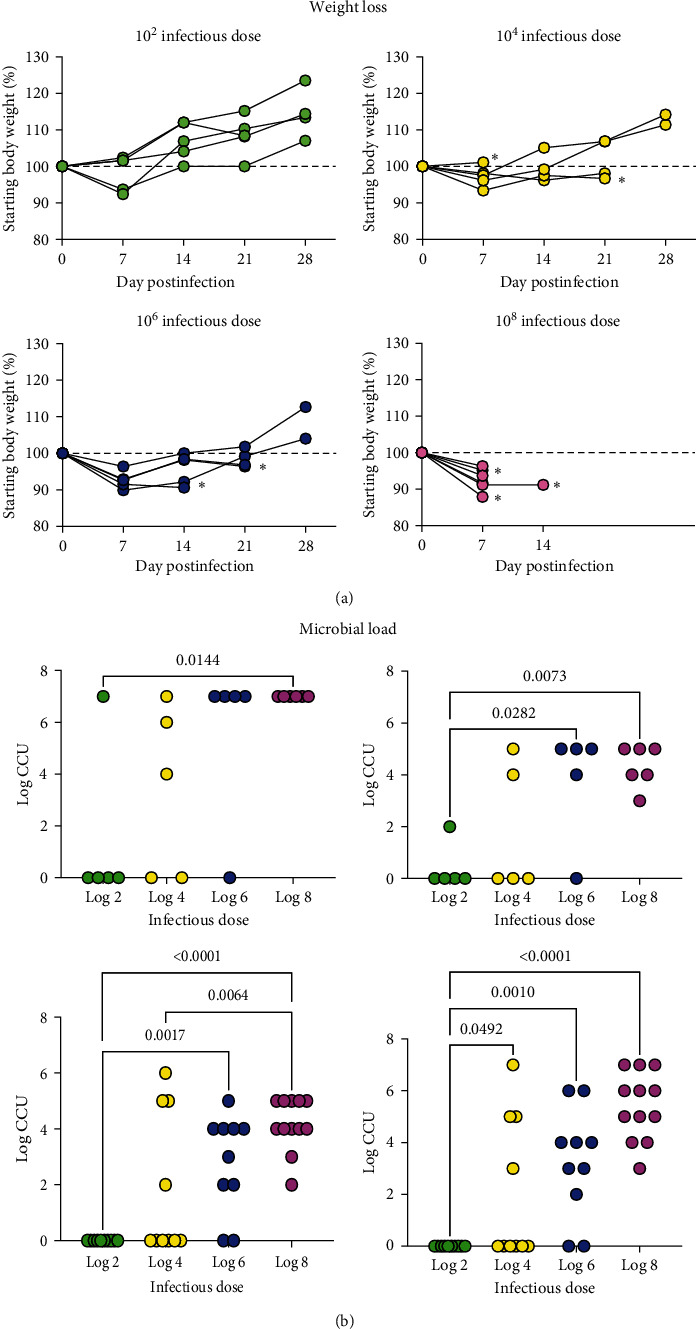
Weight loss (a) and bacterial colonization (b) in alligators infected intravenously with *M. alligatoris*. For infectious dose 10^2^, 10^4^, and 10^6^ groups, *n* = 5; for 10^8^ group, *n* = 6. Alligators were necropsied at time of euthanasia or 28 days postinfection. (a) The starting baseline weight is shown by the dashed line; all data are expressed in percentage of the starting body weight.  ^*∗*^ denotes last time point animal was weighed prior to euthanasia due to clinical criteria or mortality event. Note that in the 10^8^ infectious dose, only one animal survived to the 14-day time point. (b) Culture results are shown for blood, brain, elbow, and knee. Both left and right joint sites are shown. All data are expressed as log CCU. Data were analyzed by one-way ANOVA; significant differences among groups were determined with Tukey's multiple comparison test. *p* values < 0.05 are shown. A linear trend was found for all sites (*p*  < 0.002).

**Figure 4:**
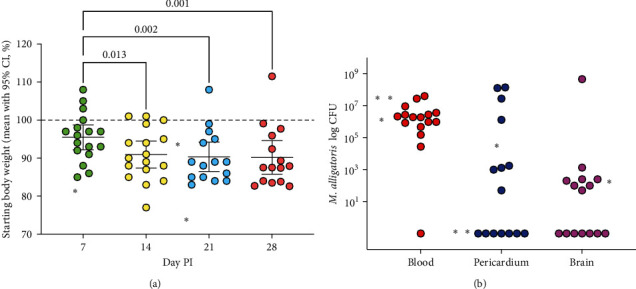
Weight loss (a) and microbial load (b) in multiple body sites following intravenous (IV) infection with 10^6^*M. alligatoris*. Three animals were euthanized at 6, 15, and 16D PI; all others were euthanized at D28 PI.  ^*∗*^ denotes the weight and microbial load of these three animals at time of euthanasia. (a) The starting baseline weight is shown by the dashed line; all data are expressed in percentage of the starting body weight. (b) Consistent bacteremia was found at necropsy with a subset of animals also infected in the brain. Note that two animals that were euthanized did not have a brain culture taken.

*G. mellonella* fifth instar larvae were purchased from a commercial distributor (Waxworms.net, Saint Marys, OH). Upon arrival, larvae were screened to remove any dead, graying/discolored, or melanized insects. Remaining larvae and bedding were disinfected with 70% ethanol and left for 24 hr at room temperature to acclimate. *Galleria* was injected in one of the last prolegs using a sterile 27G hypodermic needle (Fisher Scientific catalog# 14-841-01) and 100 *μ*L Trajan fixed Luer Syringe (Fisher Scientific #SG05229), using standardized infection protocols [67, 89]. Infection doses for *G. mellonella* were selected based on those used in the intravenous (IV) dose response study (Figures 2 and 3) of the natural host. Based on the survival curve (Figure 2), we selected 10^2^ as the low dose, as all alligators survived and 10^4^ as the medium dose. A high dose of 10^6^ compared to 10^8^ was selected based on the consistent clinical findings of the single IV infection (Figure 4). Infected larvae (*n* = 50 per infection dose) were injected with 10 *μ*L of culture; two control groups of larvae received either 10 *μ*L sterile SP4 broth (*n* = 75) or 10 *μ*L sterile phosphate-buffered saline (PBS) (*n* = 75); a third group of larvae (*n* = 75) received no injection. Following injection, larvae were individually placed into wells of a 12-well culture plate (Corning#3512) with disinfected bedding. *G. mellonella* were kept in secondary containment at 30°C for 28 days and monitored daily for mortality, pupation, and emergence.

Most infection studies using *G. mellonella* do not extend beyond the larval stage. However, insect model systems are also commonly used to test chemical compounds for toxicity [66, 69, 90–93]. These studies, like ours, extend across the life cycle and provide the criteria for scoring and determination of impact on life stage. To determine the impact of infection on the outcome of different life stages, we classified life, death, and/or arrested events. Life events were considered those in which an insect was successfully able to transition to the next stage (e.g., larval to pupal stage; pupal stage to emergence). An arrested event was when an insect remained alive for the duration of the study but did not successfully transition to the next life stage. In some cases, larvae began the transition to pupa, but never progressed and presented as larval–pupal intermediates. Death events were considered those in which an insect died in a life stage or during transition to life stage (e.g., larval–pupal intermediate). Determination of mortality was based on established health index scores [94]. In all cases, mortality was confirmed by the lack of movement after stimulation by gentle prodding with a sterile pipette tip. At the end of the study (28 days) or at time of death prior to 28 days, *G. mellonella* were placed at −20°C for a minimum of 24 hr and then autoclaved.

### 2.8. Mycoplasmal Colonization of *G. mellonella*

A separate cohort of larvae (infected and controls) were inoculated for hemolymph collection. At D1, 3, 5, and 7 PI, five larvae were sacrificed per group. Larva were chilled, hemolymph was collected by making a small incision below the last proleg and allowing the hemolymph to drain into sterile 1.5 mL tubes. In order to limit melanization prior to processing, tubes were kept on ice [95, 96]. Hemolymph (20 *μ*L) was serially diluted 10-fold in sterile SP4 broth, 20 *μ*L of each dilution was plated on SP4 agar, and plates were incubated for 72 hr at 30°C. Bacterial counts were determined by CCU and CFU. As additional confirmation, an agar plug of single colonies was obtained for PCR; for control plates, an agar plug of the primary dilution spot was used to confirm culture negative status. Total nucleic acid was extracted from the hemolymph (DNeasy blood and tissue kit #69504, Qiagen) and from agar plugs (PureLink™ Genomic DNA mini kit #K18002, ThermoFisher Scientific) following the manufacturer's instructions. PCR amplification (Jumpstart™ ReadyMix™, Millipore–Sigma# P2893) of the16S rRNA and *mscL* genes was done on extracted DNA from agar plugs to confirm isolates were *M. alligatoris*. RT-qPCR was performed on hemolymph extracts (Luna™ Universal One-step RT-qPCR kit #E30056S, New England Biolabs). Total nucleic acid from the stock culture used for inoculations served as a positive control. Primers and details of both assays are provided in Table S1.

### 2.9. Statistical Analysis

All statistics were conducted using Prism 9.0 (GraphPad Software, San Diego, CA). For alligator infection studies, data were analyzed by one-way ANOVA; significant differences among groups were determined with Tukey's multiple comparison test. Survival curves for both alligator and *G. mellonella* infection studies were analyzed by Kaplan–Meier and log-rank Mantel–Cox test for significance. Linear trend analyses were performed on dose response studies. For *Galleria* infection studies, percent larval survival/mortality, pupation, and emergence were measured for each individual life stage; larvae receiving sterile SP4 medium served as the control data set. Based on the D'Agostinos & Pearson test of normality and goodness of fit, one-way ANOVA was used for larval survival; Kruskal–Wallis was used for pupation, pupal death, and emergence. Impact of infection on melanization score was performed by Chi-square. A *p*-value of 0.05 was considered statistically significant for all tests.

## 3. Results

### 3.1. Weight Loss and Microbial Colonization Levels in Tissues Correlated with Infectious Dose in Alligators Infected Intratracheally with *M. alligatoris*

*M. alligatoris* was recovered from blood, brain, and joints in a dose-dependent manner (Table 2). At low doses, animals appeared to clear, or reduce the microbial load below detectable levels, in all tissues. At higher doses, at least one animal/infectious dose had detectable levels in blood at D14 PI. At Day 14, positive blood cultures were seen in 1/6 of animals receiving 10^2^ or 10^3^ CFU (low) infectious dose, in 3/8 receiving 10^4^ or 10^5^ CFU (medium) infectious dose, and 3/7 receiving 10^6^ or 10^7^ CFU (high) infectious dose. By D28 PI, 80% (8/10) of animals in the high dose had *M. alligatoris* in the blood as opposed to none in low dose and 30% (3/10) in the medium dose. Colonization of joints was not observed at D14 PI in any group; however, by D28 PI *M. alligatoris* was recovered from joints of 1/5 (medium dose) and 3/5 (high dose) animals. The one positive animal in the medium dose group had *M. alligatoris* recovered from three of its four joints. In the high dose group, two animals had *M. alligatoris* recovered from only a single joint while the third alligator had *M. alligatoris* recovered from all four joints.

**Figure 5:**
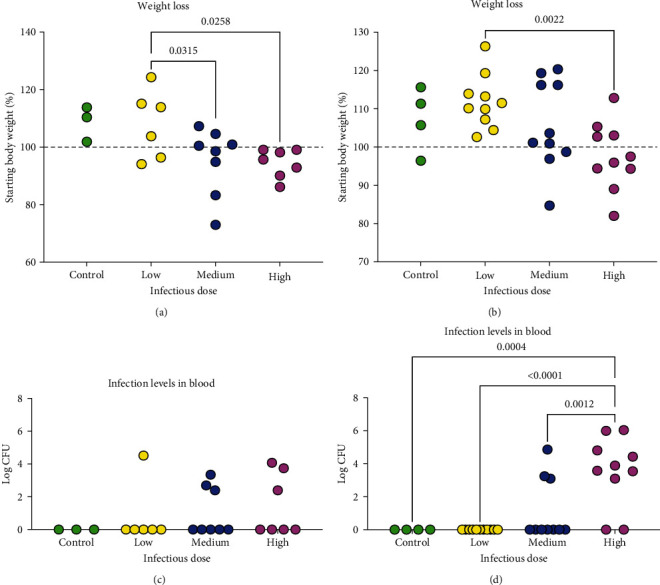
Weight loss (a, b) and bacterial colonization (c, d) in alligators infected intratracheally with *M. alligatoris*. Infectious doses used were low (10^2^ and 10^3^ CFU), medium (10^4^ and 10^5^ CFU), and high (10^6^ and 10^7^ CFU). Controls received sterile broth. Alligators were necropsied at Day 14 (control, *n* = 3; low, *n* = 6; medium, *n* = 8; high, *n* = 7) or Day 28 (control, *n* = 4; low, *n* = 10; medium, *n* = 10; high, *n* = 10) postinfection. Data were analyzed by one-way ANOVA; significant differences among groups were determined with Tukey's multiple comparison test. *p* values < 0.05 are shown. (a, b) The starting baseline weight is shown by the dashed line; all data are expressed in percentage of the starting body weight. A linear trend was found at both Day 14 (*p*=0.008) and Day 28 (*p*=0.002). Isolation of *M. alligatoris* from blood of intratracheally infected alligator is expressed as log CFU. At Day 28 PI (d), microbial load was significantly greater in the high infectious dose than all other groups, *p*  < 0.0001; there were no differences at Day 14 PI (c). A linear trend was found at Day 28 (*p*=0.0001) but not at Day 14 (*p*=0.205).

Based on the results of the microbial colonization levels (Table 2), the 10^1^ CFU infectious dose was omitted from further study. For all future analyses, infectious doses were considered as low (10^2^ and 10^3^ CFU), medium (10^4^ and 10^5^ CFU), and high (10^6^ and 10^7^ CFU). Significant loss of body weight was seen in both the medium (*p*=0.0315) and high (*p*=0.0258) necropsied at D14 PI (Figure 5(a)). All animals in the high dose were below baseline body weight at D14 PI. By D28 PI (Figure 5(b)), all animals in the low dose group were at or above their starting baseline and this group had significantly greater weight gain than did high dose animals (*p*=0.002); 70% and 40% in the medium and high dose groups, respectively, had attained or exceeded their starting baseline weight. A linear trend in increasing weight loss with increasing infectious dose was seen at both D14 (*p*=0.008) and D28 (*p*=0.002) PI.

We next compared the microbial load in blood (Figures 5(c) and 5(d), as this was the most consistent site of colonization (Table 2). A linear trend was noted for increased microbial burden with increased infection dose at D28 PI (*p*=0.0001) but not at D14 PI (Figures 5(c) and 5(d). Only one animal in the low dose group had a positive blood culture at D14 PI (Figure 5(c)); three animals in the medium and high dose groups were culture positive. Interestingly, the CFU in these seven animals was similar regardless of infectious dose. By D28 PI (Figure 5(d)), no colonization of blood was detected in animals in the low dose group and only three animals in the medium dose group had positive blood cultures. In contrast, the CFU of *M. alligatoris* recovered from blood of the high dose group was greater than all other groups (*p*  < 0.001) and all but two animals had positive blood cultures.

### 3.2. Histological Lesions Increased with Time and Infectious Dose in Alligators Infected Intratracheally with *M. alligatoris*

No lesions were seen by gross examination in the animals 2 weeks post-inoculation. The tracheal mucosal epithelium of six animals including two controls was moderately to severely hyperplastic with lymphocytic infiltrates. Mild to severe infiltrates of heterophils, macrophages, and lymphocytes and edema were present in mediastinal connective tissue near the base of the heart. Two animals in the 10^4^ CFU dose group had focal areas of lysis of brainstem white matter with gitter cell accumulation. Three other animals in the 10^1^ (*n* = 1) and 10^5^ (*n* = 2) CFU dose groups had acute focal or multifocal hemorrhage in the brainstem. The lesions at 4 weeks postinoculation included the mediastinal inflammation seen at 2 weeks, a lesser degree of tracheal mucosal hyperplasia, and rare interstitial pneumonia. At 4 weeks postinoculation, animals receiving 10^6^ CFU had fibrinosuppurative polyarthritis (*n* = 3), edema of the distal limbs (*n* = 2), fibrinosuppurative epicarditis and pericarditis (*n* = 1), and fibrinosuppurative pleuritis (*n* = 1). Slight heterophilic interstitial pneumonia was found in one animal in each of the 10^5^ and 10^6^ CFU dose groups; moderate to severe multifocal to coalescing heterophilic interstitial pneumonia was present in one animal that received 10^4^ CFU. One animal in each of the 10^2^ and 10^3^ CFU dose groups had a focal area of lysis in the neuropil of the brainstem with associated gitter cells.

### 3.3. Intravenous (IV) Infection Dose Impacted Survival of Experimentally Infected Alligators (Figure 2)

The survival of alligators differed significantly (*p*=0.0003) among infectious doses. No mortality was observed in animals receiving either 10^2^ CFU *M. alligatoris* or sterile SP4 broth. Overall survival in animals receiving 10^4^ or 10^6^ CFU *M. alligatoris* were similar and two alligators in each group survived until the scheduled necropsy at D28 PI. Of the animals receiving 10^8^ CFU *M. alligatoris*, only one survived to D14 PI.

### 3.4. Body Weight Loss Was Prolonged at Higher Infection Doses (Figure 3(a))

At D7 PI, alligators initially lost weight in all infectious dose groups, except for two animals (10^2^ CFU dose) and one animal (10^4^ CFU dose), both of which maintained their starting weight but did not gain weight. By D14 PI, all but one animal in the 10^2^ CFU dose was above the starting baseline weight. Virtually all of the animals did not meet the weight loss greater than >20% of the original body weight requiring euthanasia, but at higher infectious doses severe lameness of multiple limbs in conjunction with weight loss was observed. Prior to D28 PI, 12/21(57%) infected alligators met the criteria for euthanasia (Figure 3(a)). Euthanasia was required for animals receiving 10^4^ CFU (*n* = 1 at D7 PI; *n* = 2 at D21 PI) and animals receiving 10^6^ CFU (*n* = 1 at D14 PI; *n* = 2 at D21 PI). Clinical disease was more severe in animals receiving 10^8^ CFU; five were euthanized at D7 PI and one at D12 PI. No animals in the 10^2^ CFU dose met the criteria for euthanasia. At the D28 PI study end, all nine alligators remaining in the study were above the starting baseline weight, regardless of infectious dose.

### 3.5. Intravenous (IV) Infection Dose Correlated with Microbial Load in Multiple Body Sites (Figure 3(b))

For all sites, a linear trend was found, with microbial load increasing as infectious dose increased (*p*  < 0.002). The culture results for the elbows and knees include both right and left joints. Within each infection dose, some individual variation in clinical signs and colonization sites were seen. No animals in the 10^2^ infectious dose group had gross clinical lesions or joint colonization at necropsy (D28 PI); however, at necropsy, *M. alligatoris* was isolated from the blood and brain of one alligator. In the 10^4^ infectious dose group, two alligators had clinical signs requiring euthanasia at D21 PI; both alligators were colonized in all sites and had swelling of one or more footpads. Two animals in the 10^4^ infectious dose group did not show overt clinical signs and were necropsied at D28 PI; one animal had a positive blood culture and the other was culture negative at all sites. One animal in the 10^2^ infectious dose group was found dead at D9 PI, presumably due to trauma, and all sites were culture negative. At the higher infectious doses (10^6^ and 10^8^), *M. alligatoris* was recovered from the majority, if not all, of sites. Three of five alligators in the 10^6^ group were euthanized prior to D28 PI, one at D15 and two at D21. All three animals euthanized prior to D28 PI had *M. alligatoris* isolated from all body sites and clinical signs of foot swelling or lameness. Only two animals in the 10^6^ group were necropsied at D28 PI. Interestingly, neither animal had clinical signs of joint involvement, but one was colonized in all sites while the other was culture-negative for all sites. In contrast to animals in the other infectious dose groups, all six alligators receiving the 10^8^ infectious dose met the criteria for euthanasia within 2 weeks PI. These animals were necropsied at D11 (*n* = 2), D12 (*n* = 3), and D14 (*n* = 1) PI. *M. alligatoris* was recovered in high numbers from all body sites of all six alligators in the 10^8^ infectious dose group and all had evidence of joint involvement; one animal also had a severe fibrinous exudate in both hip joints at necropsy.

The most frequent gross lesion at necropsy was severe swelling of at least one distal limb (2/5 animals in 10^4^ group; 2/5 in 10^6^ group, and 6/6 in 10^8^ group). This swelling was characterized by accumulation of large amounts of clear gelatinous material distending the subcutaneous space of the carpal/metacarpal or tarsal/metatarsal regions, also involving the footpads and webs. One animal in the 10^8^ group also had yellowish-tan loosely adherent fibrinous exudate within both coxofemoral joints, urate deposition in the mesentery, and a 0.5 cm diameter round abdominal mass loosely attached to the root of the mesentery.

### 3.6. Intravenous (IV) Infection with 10^6^ CFU *M. alligatoris* Resulted in Sustained Weight Loss, Consistent Bacteremia, and Substantive Joint Swelling and Lameness

In the initial IV dose response study, animals were euthanized based on weight loss, joint swelling, and lameness, resulting in only two animals in the 10^6^ infectious dose and no animals in the 10^8^ infectious dose surviving to D28 PI. Some animals receiving 10^6^*M. alligatoris* were able to gain weight. To better understand the later stages of infection, we performed a second IV infection study. Alligators (*n* = 18) were infected intravenously with 10^6^*M. alligatoris*. Animals lost weight each week (Figure 4(a)), with each week's PI consistently lower than the starting baseline weight as well as the body weight at 7D PI. Three animals were euthanized at 6, 15, and 16D PI; all others were euthanized at D28 PI. Individual weight loss patterns (Figure 4(a)and Figure S1) at 28D PI fell into two main groups: those animals which were able to maintain or exceed 90% of the initial starting body weight (Figure S1a) and those which were unable to maintain 90% (Figure S1(b)), a subset of which fell below 85% of the initial body weight. At 28D PI necropsy (*n* = 15) or at day of euthanasia (*n* = 3, at D7,15, or 16 PI), *M. alligatoris* was cultured from blood (17/18, 94%), pericardium (9/18, 50%), and brain/meninges (9/16, 56%; two not tested) of alligators (Figure 4(b)). No weight loss or clinical signs were observed in any control animals.

### 3.7. Clinical Signs and Histological Lesions in Animals Infected Intravenous (IV) Infection with 10^6^ CFU *M. alligatoris* Were Consistent with Culture Results

The most consistent clinical presentation (Figure S2(a)) was foot swelling and lameness. Both swelling and lameness were recorded as total days (Figure S2(b)); for a given day, each joint was assessed separately for a total of four possible events per day. Thus, an animal could have a maximum of 4 events/day or 112 events/study if necropsied at D28 PI. Three animals required euthanasia prior to D28. One alligator necropsied at D15 PI had significant joint swelling. Based on 15 days × 4 joints assessed/day, this animal had 60 swollen joint events/60 total observations and 55 lameness events/60 total observations. No evidence of swollen joints or lameness was observed in the two additional animals that required euthanasia at D7 and D15 PI, respectively. Only one animal necropsied at D28 PI had no evidence of arthritis and joint involvement during the 28-day period. Several animals were observed scratching at the dorsal surface of their necks and heads and floating at a tilt or circling in the water. Based on histologic examination, these animals had encephalitis and meningitis. The scratching behavior was thought to be a reaction to pain or altered sensation associated with the brain lesions. Although much less frequent than joint involvement, gross pericarditis (*n* = 3) and evidence of meningitis alone (*n* = 3) or in conjunction with encephalitis (*n* = 3) also was documented.

**Figure 6:**
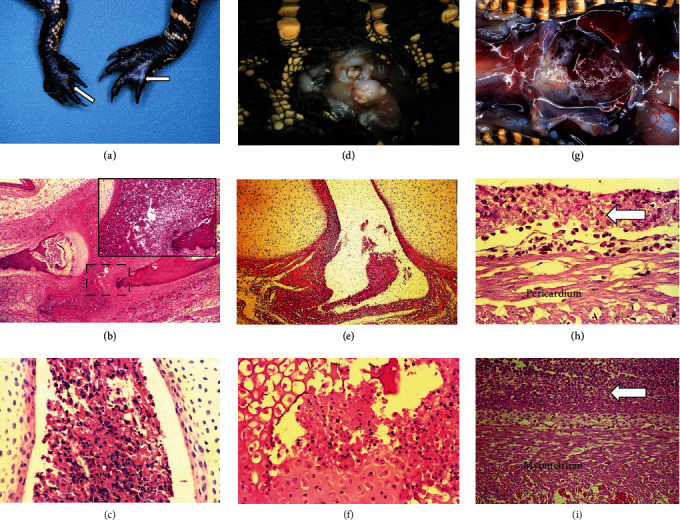
Representative gross and histological lesions in alligators infected with *M. alligatoris*. Lesions of the joints include (a) swollen footpads (arrows), (b) severe inflammation of the digit; the insert is a higher magnification of the area in hashed lines, showing infiltration of inflammatory cells and (c) inflammatory infiltrates into the joint space. Suppurative lesions of the coxofemoral joint (d) with synovitis (e) as well as occasional osteomyelitis (f). Gross fibrinous pericarditis (g) with inflammatory infiltrates (arrows) above the pericardium (h) and myometrium (i).

Histologic lesions characteristic of septicemia were observed and included fibrinosuppurative meningitis and suppurative encephalitis; polyarthritis with associated cartilage necrosis, osteomyelitis, fasciitis, tendinitis, and myositis; and fibrinosuppurative epicarditis and pericarditis. The pericarditis was fibrinopurulent with a dense layer of fibrin, granulocytes, and macrophages, and occasional multinucleated giant cells. Representative gross and histological lesion for the joints and heart are shown (Figure 6(a)–6(f)). The composite index score for each animal is shown for the joints and brain (Figure S3(a)). The individual joint lesion scores used to calculate the joint (Figure S3(b)) and brain (Figure S3(c)) composite lesion indices are provided. Severe lymphoid hyperplasia in the periarteriolar and periellipsoidal lymphoid sheaths of the spleen was present in all animals (Figure S4). Scores were based on the criteria in Table 1. The higher the index score, the more severe the lesions.

### 3.8. *M. alligatoris* Readily Colonized *G. mellonella* hemolymph (Figure S5)

Microbial load in the hemolymph increased over time (linear trend, *R*^2^ = 0.9583). By D3, the microbial load for all infectious dose groups had surpassed that of D0. *M. alligatoris* growth was rapid, and by D7 (Figure S5a) had reached 10^12^ CFU, which was the upper limit of our broth dilution. Therefore, RT-qPCR of the *mscL* gene in the hemolymph (Figure S5(b)–S5(c)) was performed as an additional confirmation of the CFU results we observed (Figure S5(a)). Infectious dose groups differed significantly (*p*=0.0251) by day and a linear trend (*R*^2^ = 0.8177) was observed (Figure S5(d)).

### 3.9. Infection with *M. alligatoris* Significantly Impacted Lfe Stage Outcome (Figure 7(a)–7(d)

**Figure 7:**
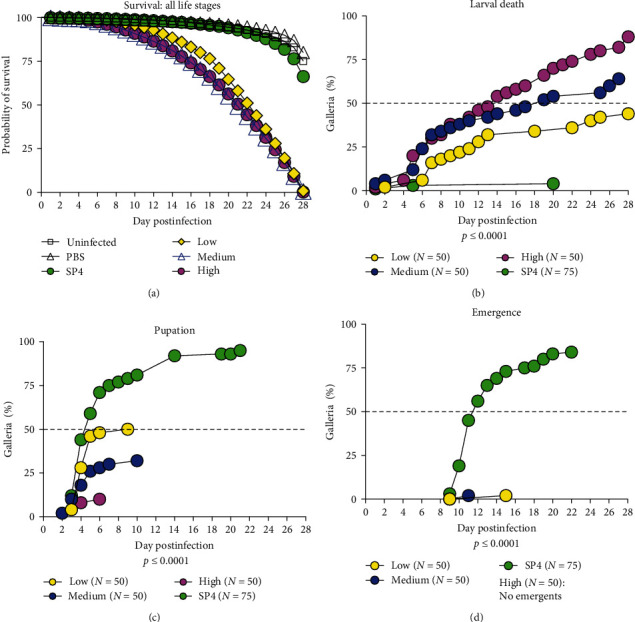
Impact of *M. alligatoris* on *G. mellonella* survival and transition across life stages. (a) Survival curves of control and *M. alligatoris* infected *G. mellonella* were significantly different (*p* ≤ 0.001, Kaplan–Meier and log-rank Mantel–Cox test). Differences in individual life stages were measured by percent larval mortality (b), pupation (c), or emergence (d). SP4-inoculated insects were the control group. (b) Larval mortality was significantly different (*p* ≤ 0.0001), one-way ANOVA. All groups were significantly different (Tukey's multiple comparisons), with the exception of larval mortality between the medium and high doses (*p*=0.1759). (c) Successful pupation among groups was significantly different (*p* ≤ 0.0001), Kruskal–Wallis test. Medium vs. SP4 (*p*=0.0074) and high vs. SP4 (*p*=0.0004) were significantly different, Dunn's multiple comparison test. (d) Emergence was significantly difference between SP4 controls and both low (*p*=0.0005) and medium (*p*=0.0028) infection doses, Dunn's multiple comparisons test. No insects in the high dose group were able to emerge.

The overall survival proportions for *G. mellonella* inoculated with *M. alligatoris* were significantly different from control insects (Figure 7(a), *p*  < 0.001). Survival curves (Figure 7(a)) and emergence rates (Figure S6) were not different for insects receiving SP4 medium, PBS, or no intervention. Therefore, SP4 medium was used as the control data set for data analyses. Specific differences in impact of infection were best seen in individual life stages. *G. mellonella* infected with *M. alligatoris* had lower survival proportions across all three life stages (larva, pupa, and emergent) compared to control insects (Figure 7(b)–7(d)). Mortality in SP4 controls was low (4%) in the larval stage (Figure 7(b),*p*  < 0.0001). Conversely, larval mortality in infected insects was high and dose dependent, ranging from 50% (low dose) to 90% (high dose). Because larvae infected with *M. alligatoris* were arrested and unable to transition to pupa, the larval mortality events occurred throughout the 28-day period. Successful transition to pupae occurred in 95% of SP4 control larvae; however, pupation of *G. mellonella* infected with *M. alligatoris* was severely impacted and dose dependent ((Figure 7(c), *p*  < 0.0001). The highest successful pupation rate was in the low dose group at only 50%. Overall successful emergence (Figure 7(d)) occurred in 84% of SP4 controls, with only one successful emergence in either low or medium dose infection groups (*p*  < 0.0005). No high dose insects completed the transition to emergence.

### 3.10. Infection with *M. alligatoris* Significantly Impacted Life Stage Timing (Figure S7)

The normal progression for developmental life stages was seen in SP4 controls, where 95% of larvae successfully underwent pupation (Figure S7(a)). Beginning at D3, control larvae began transitioning to pupa, with peak pupation reached between D5 (50%) and D7 (75%). By D14, 95% of the larvae had transitioned to pupa; only 5% were unable to transition. Emergence occurred in 84% of *G. mellonella*, beginning at D9 and ending at D22. Emergence was incremental, with 50% at D12 and 75% at D17. Pupal deaths were limited to 10%, occurring primarily after D21. In direct contrast to the sharp bell curve associated with pupation in SP4 controls, *G. mellonella* infected with *M. alligatoris* at low and medium doses had curves which were lower, flattened, and spread across a wider time frame (Figure S7(b)–S7(d)). The successful transition from larva to pupa was only 50% (low dose, Figure S7(b)) and 32% (medium dose, Figure S7(c)). Initial pupation began at the same time as SP4 controls; however, the curves were flattened and accompanied by increasing mortality in the pupal stage beginning at D9 (medium dose, Figure S7(c)) and 12 (low dose, Figure S7(b)); only one emergent was seen in either low or medium dose groups. All *G. mellonella* infected with the high dose (Figure S7(d)) died in either the larval (90%) or pupal (10%) stages.

### 3.11. Melanization Scores at Time of Death Differed Significantly between Control and Infected Insects (Figure 8)

Melanization is a defense mechanism to trauma or infection and can be a direct visual indication of the impact of infection in *G. mellonella*. Melanization index scores [94] were recorded at time of death for larval, intermediate, and pupal life stages (Figure 6(a)–6(f). Scoring distributions were different among the groups, *p*=0.044, Chi-square. Except for one SP4 control insect (score = 2), all controls that died were either fully melanized (score = 0) or had no melanization at time of death (score = 4). In contrast, infected groups had a wider range of melanization scores, with all scores represented. Compared to the high dose group, low and medium infectious groups had higher percentages of intermediate (2 or 3) or no melanization (4) scores. Within groups, there was a significant difference between scores of low (*α* = 0.0041) and medium (*α* = 0.0356) infectious dose groups. There was a significant difference in insects that were either not melanized (*α* = 0.0149) or fully melanized (*α* = 0.0467). Additionally, a dose response was observed for infected insects that were fully melanized with numbers increasing with infectious dose. In addition to melanization scoring, larval–pupal intermediates were recorded at time of death. Intermediate life stage events are often a result of disruption due to trauma, toxicological, or pathological insults during transition to the next life stage [97]. A small group of infected insects (*n* = 5, low; *n* = 8, medium; *n* = 6, high) presented as larval–pupal intermediates (Figure 7(c)) at death; no larval–pupal intermediates were seen in SP4 control insects.

## 4. Discussion

**Figure 9:**
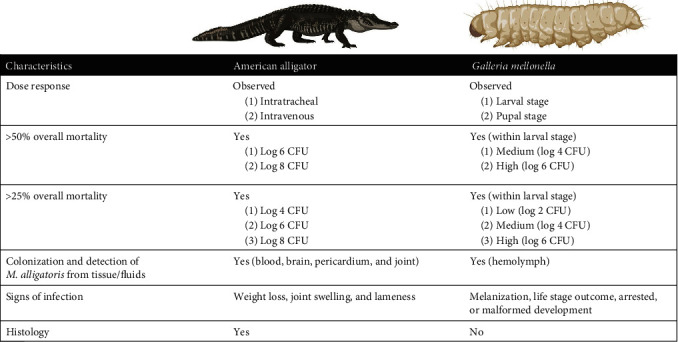
Comparison of outcomes following experimental infection of the American alligator and *G. mellonella*. Overall mortality for alligators is defined as animals that died spontaneously or met euthanisa criteria. Overall mortality for *Galleria* is defined as death across all life stages; insect mortality was confirmed by the lack of movement after stimulation by gentle prodding with a sterile pipette tip.

The goals of this current study were to expand the initial experimental infections with *M. alligatoris* that fulfilled Koch's postulates to better understand the disease in the natural host and to determine if the invertebrate model *G. mellonella* could serve as a surrogate alternate host. A comparison between the natural and invertebrate hosts showed striking similarities with respect to the outcome of the dose response infections (Figure 9). A dose response to infection was observed in both alligators and *Galleria*. In the alligator, >50% mortality was observed at both the 10^6^ and 10^8^ CFU infectious doses, with <25% mortality at 10^4^ CFU. Within the *Galleria* larval stage, >50% mortality was observed at both 10^6^ and 10^4^ CFU, with >25% mortality seen at 10^2^ CFU. *M. alligatoris* was able to colonize and grow well in both alligators and *Galleria*. Clinical signs of infection were seen in both alligators (weight loss, joint swelling, and lame) and *Galleria* (melanization, life stage outcome and days to life stage transition, and malformed larval/pupal intermediates).

Mycoplasmal infection usually presents as a chronic, clinically silent disease with high morbidity but low mortality [2, 4–7, 9, 22–26]. In contrast, during the initial 1995 epizootic of captive American alligators, mortality was rapid with >80% of the population dying within a 6-month period [29]. Moreover, histological examinations confirmed that there was multisystem involvement with pneumonia, endocarditis, arthritis, and myocarditis [27]. The route of *M. alligatoris* transmission in wild populations is not known, but based on what is known for other mycoplasmal infections, direct contact or aerosol transmission is assumed likely. During mating season [98], the bellowing of males could create significant aerosols. Mating season is also associated with aggressive behavior which would result in more direct contact. No disease outbreaks have been observed in wild populations, but there is serological evidence to support exposure in select wild populations [28, 99]. Notably, in the initial outbreak, dead animals were buried in shallow graves in the enclosure which had been depopulated. New naive animals were then released into the area. Not unexpectedly for predators, cannibalism occurred, resulting in a subsequent outbreak. In the wild, this may be a significant source of infection as this behavior is typical; animals with arthritis and limited mobility would be easy prey. Environmental transmission is less likely, as *M. alligatoris* does not grow well above 30°C and in fact dies rapidly at 37°C [87]. Transmission via vectors is also unlikely and has rarely been documented for *Mycoplasma* spp. Vector transmission of the nonculturable hemotropic mycoplasmas, which preferentially attach to erythrocytes but are not associated with other mucosal or systemic body sites, can occur [17–20, 100]. Although insects are well-documented to transmit *Phytoplasma* and *Spiroplasma* spp. [101–103], these genera are not associated with infections in vertebrate hosts.

In the natural host, the American alligator, we initially used the IT route of infection because that was the original route used in early infection studies [27, 29, 41]. We also used the results from these studies to inform our choice of infectious doses. Briefly, in the original study to prove Koch's postulates [29], 10^6^ CFU of *M. alligatoris* were introduced into either the trachea via the glottis or into the intracelomic cavity by injection. Although only two animals were used in each group, three of four died within 3 weeks. Subsequently, a dose response study [27] was performed using 10^2^, 10^4^, or 10^6^ CFU and the IT route of infection. The clinical presentation was similar to that observed during the natural epidemic which occurred in 1995. One of the goals of this early study was to determine the antibody response, but 50% of animals receiving 10^6^ CFU died before an antibody response could be detected [27]. As in our study, pericardium and heart (blood) had the highest microbial load, even though the initial route of infection targeted the respiratory tract.

In the current study, IT infection of the alligators revealed *M. alligatoris* preferentially disseminated to the blood, brain, and joints of infected animals. The high level of bacteremia and multisystem colonization by *M. alligatoris* was an unexpected finding. Mycoplasmas typically display an affinity for mucosal surfaces, inhabiting sites such as the respiratory and genital tract [10, 21, 24]. Although spread to extrapulmonary and nongenital sites, including the joints and nervous system can occur [4, 8, 104–111], it generally is not as common. Similarly, isolation from the blood is rare with the exception of the nonculturable hemotropic mycoplasmas which preferentially attach to erythrocytes but are not associated with other mucosal or systemic body sites [17–20, 100, 112].

Because the most dominant site of colonization in the IT-infected animals was blood, we sought to determine the impact of IV infections on clinical outcomes. The infectious doses used in the IV infection experiments spanned the range of those used in the current IT studies as well as in the early experiments with limited numbers of animals [27]. Although the IT infection study showed the ability of the pathogen to colonize extrapulmonary sites, the IV study more fully reflected the pathogenicity seen in the natural infection.

In the natural infection, animals died rapidly and in good flesh. A subset also had neurological disease (meningitis and encephalitis) and were culture positive in the brain and meninges. The older life stage of the population was considered as a possible factor of disease severity, including spread to the brain. However, the experimental infections to fulfill Koch's postulates and establish *M. alligatoris* as the etiologic agent was performed in young adult alligators, and the same clinical signs were observed [27]. Our current study confirms that pathogenicity is not affected by age, as both 12- and 22-week-old juvenile alligators infected with *M. alligatoris* had the same clinical outcome as the naturally infected alligators, displaying lameness and swelling in the joints, microbial colonization of multiple tissue sites, and mortality in individuals that received a higher dose. Additionally, when the time of infection was extended, clinical disease was prolonged, weight loss was sustained over time, and bacteria persisted within vulnerable tissue sites (blood, brain, and pericardium). These findings helped to reveal the clinical progression and acute pathogenicity of *M. alligatoris*.

Importantly, *G. mellonella* was able to recapitulate the virulence potential and mirrored the survival curves seen in IV dose response infections in the natural host. For alligators, the most common clinical signs were weight loss and lameness/joint swelling. For insect models, the major clinical signs are mortality within a life stage or delayed or aberrant transition throughout the life stages (Figure 7). These signs are measured by days to death in the life stages, overall percent death in the larvae and across stages. Using these invertebrate-specific clinical signs, we observed similar dose response effects to those shown in the alligator. Within infected *Galleria* groups, we observed lower pupal and emergence numbers over time as a result of death within the larval and pupal stages in a dose-dependent manner. Only a single emergence event occurred in low- and medium-infected groups, while no high-dose infected insects were able to emerge. The time required to make the successful transition is also a key clinical sign. During normal development, 50% of larvae transitioned to pupae by D4, with 80% transitioning by D10. Only 50% of larvae infected with the low dose successfully transitioned to pupae and the time required was 6 days. Larvae receiving the medium and high infection doses were essentially unsuccessful, and those few larvae that were successful required a longer time period to make the transition. Many major microbial studies using *G. mellonella* to assess pathogenicity measure only the larval stage [71, 82, 89, 94, 113–116]. This is likely due to the increased and rapid mortality of larvae in these studies, with death occurring in a matter of hours with some pathogens. Because the *M. alligatoris* study extended 28 days PI, changes in normal life stage trends were identified. Our results closely paralleled insect model systems used to test chemical compounds for toxicity [66, 69, 90–93] in that infection with *M. alligatoris* also impacted more than one life stage (larval and pupal) of *G. mellonella* in a dose-dependent manner. While larval death did occur, some larvae infected with *M. alligatoris* could initiate the transition to the pupal stage but were unable to complete the transition, resulting in the occurrence of an abnormal larval/pupal intermediate stage. One limitation of the Galleria model is the absence of an adaptive immune response. However, melanization reflects the direct innate immune defense response to pathogen or damage associated molecular patterns. Therefore, scoring the degree of melanization served as a clinical sign to determine the magnitude of the innate immune response [73–75, 83, 86, 117, 118].

Based on direct culture and qPCR, all infectious dose groups showed high levels of growth of *M. alligatoris* in the hemolymph at Days 3, 5, and 7 PI (Figure S7). D7 is the time point where 80% of control larvae entered pupation (Figure S5a) but only 10%–50% of infected larvae underwent pupation (Figure S5(b)–S5(d)). Colonization of pupae was not determined in the current study. Once pupation occurs, sacrificing the pupa prior to death would mean that the overall emergence outcome could not be determined. Given the low number that entered pupation, we deemed that it was more important to assess emergence. Most mortality events occurred during the larval stage. The additional impacts on pupation and emergence are intriguing and suggest that the *Galleria* model also might be amenable to study pathogenicity of more chronic mycoplasmal infections.

The findings of the invertebrate model have broader impacts on virulence testing of mycoplasmas. Mycoplasmas are increasingly being isolated from wildlife species and have been associated with disease outbreaks in populations of wild ruminants and various avian species [23, 50–52, 54–56, 119, 120]. In the case of *M. gallisepticum*, researchers were able to utilize captive-raised house finches to determine variations in virulence potential from the domestic strain and various North American house finch strains [53, 62, 63, 121–123]. However, not all host species are as accessible. While limited experimental infections with *M. ovipneumoniae* in big horn sheep populations have been done [124, 125], determining the pathogenic potential of strains has been difficult to conduct due to subpopulations of the natural host being listed as endangered. Novel *Mycoplasma* spp. have been isolated recently from marine mammals and reptiles [51, 59–61, 126–130], many of which are threatened and/or endangered groups and intractable for experimental infections needed to establish pathogenicity. Thus, there is a critical need for the development of alternative models to address the potential role of new *Mycoplasma* spp. in both mammalian and nonmammalian wildlife hosts.

## 5. Conclusions

The findings of this study have broader impacts on virulence testing of mycoplasmas. We propose that *G. mellonella* is a highly tractable model system to overcome the inherent limitations of establishing pathogenic potential of mycoplasmas, especially those isolated from hosts that are unlikely to be available for experimental infections. Our results support that *G. mellonella* is an excellent surrogate model system for the natural host and has the potential to expand virulence testing to other newly described *Mycoplasma* spp. from exotic and wildlife species where infection studies in the natural host are not feasible.

## Figures and Tables

**Figure 8:**
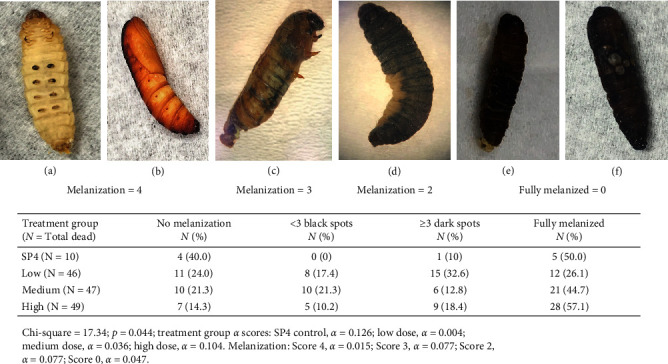
Melanization of *G. mellonella* larva or pupae at time of death. Standard scoring for melanization: no melanization, score = 4; <3 black spots, score = 3; ≥3 dark spots, score = 2; fully melanized, score = 0. Melanization scores for mortality events are shown in the table and differed among groups, *p*=0.04, Chi-square. (a) Healthy larva; (b) healthy pupa; (c) larval–pupal intermediate; (d) larva; (e) larva; and (f) pupa.

**Table 1:** Histologic criteria for assessment of lesion severity.

Site	Score	Description
Joints		

Cartilage	0	Normal
	1	Minimal focal or multifocal erosion of the most superficial layer of cells of articular cartilage
	2	Mild focal or multifocal erosion (5–6 cell layers thick)
	3	Moderate focal or multifocal erosion one-fourth or more of thickness of articular cartilage
	4	Marked focal or multifocal erosion extending to subchondral bone

Subchondral bone	0	Normal
	1	Minimal evidence of osteoclast remodeling only
	2	Mild fibrous proliferation within marrow cavity and osteoclast remodeling
	3	Moderate fibrous proliferation throughout marrow cavity and osteoclast remodeling
	4	Marked destruction of bone with osteonecrosis and osteomyelitis

Exudate in joint spaces	0	None
	1	Minimal (rare) granulocytes and mononuclear cells
	2	Mild accumulations of granulocytes, mononuclear cells, fibrin, and necrotic debris
	3	Moderate accumulations of granulocytes, mononuclear cells, fibrin, and necrotic debris
	4	Marked accumulations of granulocytes, mononuclear cells, fibrin, and necrotic debris

Synovium/fibrous capsule	0	Normal
	1	Minimal (rare to occasional) granulocytes and macrophages in synovium/fibrous capsule
	2	Mild accumulations of scattered granulocytes and macrophages in synovium/fibrous capsule
	3	Moderate, dense accumulations of granulocytes, and macrophages limited to fibrous capsule ± edema
	4	Marked destruction of fibrous capsule with infiltration into adjacent soft tissues

Brain		

Meningitis	0	No lesions
	1	Mild focal meningitis
	2	Moderate focal or mild to moderate multifocal meningitis
	3	Focal granulomas associated with meninges
	4	Severe focal or moderate to severe multifocal meningitis

Encephalitis	0	No lesions
	1	Mild focal encephalitis with infiltration of scattered heterophils and macrophages in neuropil. Minimal tissue destruction
	2	Moderately dense infiltrates of heterophils and macrophages with associated tissue destruction
	3	Severe focal or multifocal encephalitis with dense infiltrates of heterophils and macrophages and associated severe destruction of tissue

**Table 2:** Isolation of *M. alligatoris* from tissues of alligators infected intratracheally.

Infectious	Blood^†^		Brain^†^		Joints^†^	
Dose	Day 14 PI	Day 28 PI	Day 14 PI	Day 28 PI	Day 14 PI	Day 28 PI
Control	0/3	0/4	0/3	0/4	0/3	0/4
10^1^	0/4	0/5	0/4	0/5	0/4	0/5
10^2^	0/3	0/5	0/3	0/5	0/3	0/5
10^3^	1/3	0/6	1/3	0/6	0/3	0/6
10^4^	0/4	2/5	0/4	1/5	0/4	1/5^‡^
10^5^	3/4	1/5	0/4	0/5	0/4	0/5
10^6^	1/3	5/5	0/3	1/5	0/3	3/5^§^
10^7^	2/4	3/5	0/4	0/5	0/4	0/5

^†^Necropsies were performed on infected alligators at D14 PI (*n* = 4) or D28 PI (*n* = 5); control alligators (*n* = 7) were necropsied at D14 PI (*n* = 3) or D28 PI (*n* = 4). Note that the total number of alligators used for each infection dose was 9; however, two infected animals (10^2^, *n* = 1; 10^6^, *n* = 1) were euthanized prior to Day 14 and therefore are not included in the table. The number of alligators with positive cultures for each site at Day 14 or Day 28 PI/total number of necropsied at that time point is provided. ^‡^*M. alligatoris* was recovered from three of its four joints cultured. ^§^*M. alligatoris* was recovered from all four joints of one alligator and from only one joint of two alligators.

## Data Availability

*M. alligatoris* ATCC 700619 strain A21JP2^T^ is available from the American Type Culture Collection. The master record for the *M. alligatoris* A21JP2^T^ genome is GenBank accession no. NZ_ADNC01000000. Additional data supporting the findings of this study are available in the supplementary material or from the corresponding author upon reasonable request.

## Supplementary Materials

Supplementary Materials Table S1: primers and conditions for amplification of 16S rRNA and mscL. Figure S1: individual weekly body weight loss following intravenous infection with 106 M. alligatoris. Figure S2: alligators infected intravenously with 106 CFU M. alligatoris had significant swollen joints and lame limbs. Figure S3: lesion severity indices for joints and brain of alligators infected intravenously with 106 M. alligatoris. Figure S4: lymphocyte proliferation in the spleen. Figure S5: M. alligatoris readily colonized G. mellonella hemolymph in a dose dependent manner. Figure S6: emergence in control larvae receiving SP4 medium, PBS, or no injection. Figure S7: infection with M. alligatoris significantly impacted life stage timing.
