# Supplementary material for: Galleria mellonella Invertebrate Model Mirrors the Pathogenic Potential of Mycoplasma alligatoris within the Natural Host
Source: Transbound Emerg Dis. 2024 Mar 22;2024:3009838. doi: 10.1155/2024/3009838 (PMC12017031; doi:10.1155/2024/3009838)
Supplement: Supplementary Materials — Table S1: primers and conditions for amplification of 16S rRNA and mscL. Figure S1: individual weekly body weight loss following intravenous infection with 106 M. alligatoris. Figure S2: alligators infected intravenously with 106 CFU M. alligatoris had significant swollen joints and lame limbs. Figure S3: lesion severity indices for joints and brain of alligators infected intravenously with 106 M. alligatoris. Figure S4: lymphocyte proliferation in the spleen. Figure S5: M. alligatoris readily colonized G. mellonella hemolymph in a dose dependent manner. Figure S6: emergence in control larvae receiving SP4 medium, PBS, or no injection. Figure S7: infection with M. alligatoris significantly impacted life stage timing. [file 3009838.f1.docx]

**SUPPLEMENTAL MATERIAL**

**Figure S1.** Individual weekly body weight loss following intravenous infection with 10^6^ *M. alligatoris.* The blue line indicates the initial starting body weight; the red line, 90% of initial starting body weight; and black line, 85% of initial starting body weight. At 28D PI, two main groups were noted. (a) Animals were able to maintain or exceed 90% of the initial starting body weight at all time points. Also shown in (a) are 3 animals, denoted by clear symbols, that had initial body weight loss of >85% in the first week and required euthanasia at or before D14. (b) All animals lost weight at 1 week PI and were unable to maintain 90% of the initial starting body weight. A subset fell below 85% of the initial body weight but did not meet criteria for euthanasia.

**Figure S2.** Alligators infected intravenously with 10^6^ CFU *M. alligatoris* had significant swollen joints and lame limbs. Each data point represents a single alligator at 28 days post infection. (a). Typical lameness posture (b). The total number of days with swollen joint or lame limb days is shown. The maximum possible days is 112 (28 days of infection X 4 sites assessed/day). Only one infected animal had no swollen or lame joints. No swollen joints or lameness was detected in any control animals (data not shown).

**Figure S3. Lesion severity indices for joints and brain of alligators infected intravenously with 10^6^ *M. alligatoris*.** Joint lesions in all four tarsal and carpal joints were scored based on cartilage erosion, subchondral bone lesions, joint space exudate, and synovitis. Brain lesions were assessed based on severity of encephalitis and meningitis. An index score was then calculated by dividing the total lesion score by the total possible points. Higher index scores are indicative of more severe lesions. The maximum score available is 1. The full scoring system is available in Table 1. (a) The combined overall lesion index for the combined scores of joints (b) and the brain (c). Each point represents an individual animal. (b) The joint lesion index scores for each joint that were used to determine the overall index score in (a). Each point represents an individual animal. Animals (n = 3) that were euthanized before the 28 day period did not exhibit arthritis and therefore were excluded to avoid bias. (c). The brain lesion index scores for encephalitis and meningitis that were used to determine the overall brain index score in (a). Each point represents an individual animal. Animals (n = 3) that were euthanized before the 28 day period were included as the criteria for euthanasia were clinical signs of nervous system disorders. Three animals had lesions consistent with both meningitis and encephalitis; three animals had meningitis in the absence of encephalitis.

**FIGURE S4.** Lymphocyte proliferation in the spleen. (a) Normal spleen from age-matched uninfected control. (b). Spleen from infected alligator showing lymphocyte proliferation and a score of 3. Central arteriole is denoted by arrow; a periarteriolar lymphoid sheath (PALS) is shown in the box. The spleens were assessed by scoring the small lymphocytes within the periarteriolar and periellipsoidal lymphoid sheaths (PALS and PELS, respectively). Scoring scale: 1 = spleens with little to no PALS and PELS; 2 = spleens with some distinct and some indistinct lymphoid sheaths; 3 = spleens with distinct lymphoid sheaths around most vessels.

**Figure S5.** *M. alligatoris* readily colonized *G. mellonella* hemolymph in a dose dependent manner. (a) Microbial load in the hemolymph increased over time (linear trend, R^2^ = 0.9583). By D3, the microbial load for all infectious dose groups surpassed that of D0. *M. alligatoris* growth was rapid, and by D7 had reached the upper limit of our broth dilution (10^12^ CFU). (b, c). The increased microbial load over time was confirmed by RT-qPCR of *mscL* at D1 PI (b) and D7 PI (c). Infectious dose groups differed significantly (p = 0.0251) by day and a linear trend (R^2^ = 0.8177) was observed (d).

**Figure S6**. Emergence in control larvae receiving SP4 medium (84%), PBS (88%) or no injection (91%) was not statistically different (Kruskall-Wallis, P = 0.0621). In contrast, only 2% of larva receiving either the Low or Medium infectious dose successfully emerged. None of larva receiving the High infectious dose successfully emerged.

**Figure S7. Infection with *M. alligatoris* significantly impacted life stage timing.** (a). The normal development of *G. mellonella* can be seen as the successful transition of larva (blue) to pupa (green) to emergent (purple). The total number of *G. mellonella* is shown on the X axis: SP4 control, N = 75; for all infection groups, N = 50. Mortality events at both larval and pupal stage (red) can occur. Deaths in the emergent stage are failure to emerge or mortality at emergence. Colored circles below the normal insect life stage images correlate with colors used in the life stage graphs (B-E). Larval numbers (blue) decrease over time due to either a successful transition to pupal stage or a mortality event. (b). SP4 controls (N = 75) show a normal life stage progression over time. Larval numbers decrease as a result of pupation (green). Pupal numbers decrease as a result of emergence (purple). Low larval and pupal mortality occurred in SP4 (b), as reflected by slightly lower final emergence numbers as compared to the starting larval numbers (N = 75). (c, d, e). Within infected groups (N = 50 for each dose), we observed lower pupal and emergence numbers over time as a result of death within the larval and pupal stages (red). Only a single emergence event occurred in low (c) and medium (d) infected groups, while no high dose (e) infected insects were able to emerge. For larval mortality, see Figure 7b.

Table S1. Primers and conditions for amplicfication of 16S rRNA and *mscL*.
